# Tip, torque and rotation of maxillary molars during distalization using Invisalign: a CBCT study

**DOI:** 10.1186/s12903-024-04529-7

**Published:** 2024-07-15

**Authors:** Dina Elfouly, Nadia M. El-Harouni, Hanan A. Ismail, Tarek El-Bialy, Ahmed Ghoneima

**Affiliations:** 1 Department of Orthodontics, Faculty of Dentistry, Alexandria University, Champollion St, P.O. Box 21521, Azarita, Alexandria Egypt; 2 Faculty of Medicine and Dentistry, University of Alberta, Edmonton, Alberta, Canada; 3 Hamdan Bin Mohammed College of Dental Medicine, Mohammed Bin Rashid University of Medicine and Health Sciences, Dubai, UAE; 4 Adjunct Faculty, Department of Orthodontics and Oral Facial Genetics, Indiana University School of Dentistry, Indianapolis, IN USA

**Keywords:** Molar distalization, Clear aligner, Angulation, Inclination, Rotation

## Abstract

**Background:**

Desirable molar distalization by bodily movement is challenging and can be difficult to achieve. This study investigated changes in molar angulation (mesiodistal tipping), molar inclination (buccolingual torque) and rotation during distalization using clear aligner therapy (CAT).

**Materials and methods:**

This retrospective study included 38 cone beam computed tomographic images (CBCTs) taken for patients treated with molar distalization using CAT. The study evaluated pre- (T0) and post-treatment (T1) CBCTs of 19 adult patients (36.68 ± 13.50 years) who underwent maxillary molar distalization using Invisalign^®^ aligners (Align Technology, Inc., San José, CA, USA) with a minimum of 2 mm distalization. Changes in maxillary molar tip, torque and rotation were measured for 61 molars (183 roots). Paired t-test was used to evaluate the differences between pre- and post-treatment readings. The level of significance was set at *p* ≤ 0.05. The reproducibility of measurements was assessed by the intraclass correlation coefficient (ICC).

**Results:**

Molar angulation did not show significant change after distalization (*p* = 0.158) however, there was significant increase in buccal molar inclination (*p* = 0.034) and mesiobuccal molar rotation (*p* < 0.001).

**Conclusion:**

Molar distalization of 2 mm did not cause significant molar tipping. Maxillary molars showed significant buccal inclination (increased torque) and mesiobuccal rotation after distalization.

## Introduction

Clear aligner therapy (CAT), a system of sequential removable plastic oral appliances, is designed to deliver small amounts of orthodontic tooth movement per aligner. Align Technology, Inc. (San José, CA, USA) has shown substantial development in clear aligners treatment in recent years, with millions of patients being treated with Invisalign around the world [1]. Due to its superior aesthetics [2] and comfort [3], patients choose clear aligners therapy as compared to conventional fixed appliances. A single aligner can provide 0.25 to 0.33 mm of targeted tooth movement every 14 days with 22 h of daily wear, however recently it was recommended to change these aligners every week [4]. The use of clear aligners was first recommended as a treatment option for minor crowding or space closure. Recently, clear aligners have been used successfully in a number of challenging cases, including extractions, open bites, deep bites, Class II malocclusion [5], and molar distalization [6].

Three-dimensional (3D) control of tooth movement is of crucial importance in orthodontic treatment. Since molar distalization is frequently used in contemporary orthodontic therapy, the amount of molar tipping must be considered during treatment. Conventional orthodontic appliances that are currently used for molar distalization like distal jet achieved 3.2 mm of maxillary first molar distalization per side, with 3.1° of distal crown tipping [7]. A systematic review by Antonarakis and Kiliaridis [8] concluded that the use of noncompliance intramaxillary appliances produce distal molar movement with a mean of 2.9 mm with an associated 5.4° of distal tipping. Another systematic review regarding conventional versus skeletal anchorage devices for molar distalization using fixed orthodontic appliances reported molar distalization with tipping in both groups [9]. Moreover, molar tipping was also expressed during distalization using CAT. A systematic review by Rossini et al. [10] concluded that 1.5 mm molar distalization using CAT does not cause significant molar tipping. Moreover, based on a cephalometric study, Ravera et al. [11] proposed that 2.25 mm maxillary molar distalization can be accomplished without considerable molar tilting.

Bolla et al. [7] and Kinzinger et al. [12] observed that fixed orthodontic appliances used for maxillary molar distalization showed buccal crown inclination. Studies involving distalization with Distal jet, Pendulum appliance, and Jones jig concluded that distal rotation around the palatal roots of the molars is a common occurrence [7, 12–14]. Most studies addressing 3D movements in CAT were not performed on cone beam computed tomography (CBCT), and typically used lateral cephalometric x-rays or dental casts that have been reported to have some limitations in their data interpretation. However, the precise methods in which the molar might move during CAT distalization have not been documented in the literature, such as changes in molar torque and rotation. Thus, the current study was designed to investigate changes in molar angulation (mesiodistal tipping), molar inclination (buccolingual torque) and molar rotation during distalization using clear aligner therapy (CAT). Null Hypothesis: Maxillary molar distalization using Invisalign has no significant effect on molar angulation, inclination nor rotation.

## Materials and methods

This retrospective study was performed on a sample of pre-treatment (T0) and post-treatment (T1) records of CBCT scans of orthodontic patients treated by the same orthodontist at a single center in Alberta, Canada. Sample size was estimated based on 95% confidence level to detect difference in molar tipping after clear aligners. Ravera et al. [15] reported that the mean difference and 95% confidence interval (CI) in molar tipping= -1.64 (-4.67, 1.39), and − 2.64 (-5.37, 0.06) for the 1st and 2nd molars, respectively. The minimum required sample size, using paired t-test, was calculated to be 16 patients, increased to 19 to make up for procedural problems. Sample size was calculated using MedCalc Statistical Software version 19.0.5 [15].

Pre-treatment (T0) and post-treatment (T1) CBCTs of 19 orthodontic patients (6 males and 13 females, mean age 36.68 ± 13.50 years) treated with orthodontic molar distalization using Invisalign were collected. All the patients were treated between the years 2018–2021. A total of 61 maxillary molars (30 first molars and 31 s molars) (183 roots) were included in this study. All CBCT images were acquired using the same CBCT machine (i-CAT, Imaging Sciences International (ISI), PA, USA), and the settings used were in accordance with manufacturers’ recommendations (8.9 s, 13 × 16 cm FOV, 120 k, 10 mA and 360° rotation, and voxel size 0.3 mm). All images were acquired with the subjects’ heads positioned such that the Frankfort horizontal plane ran parallel to the floor. Images were saved as digital imaging.

**Fig. 1:**
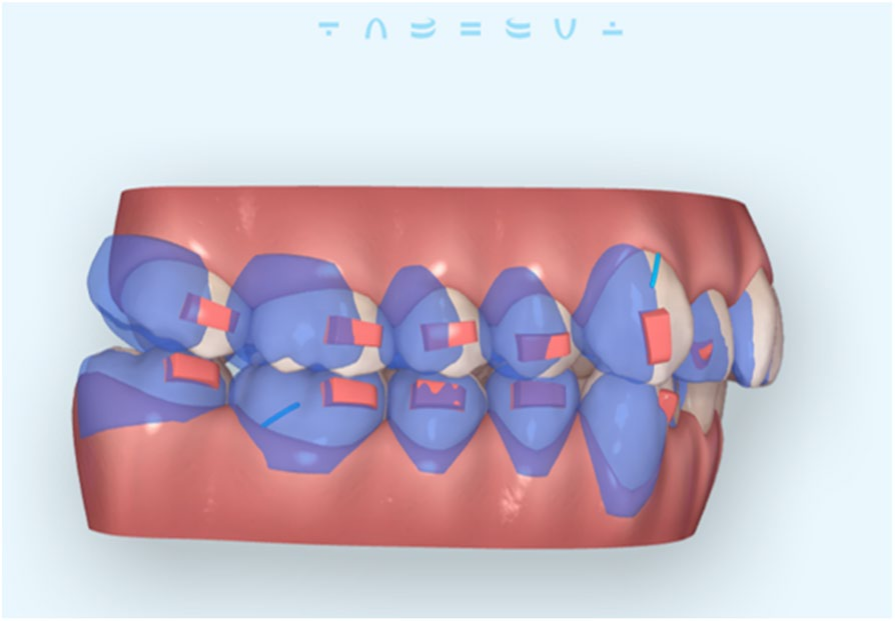
Illustration of the used mechanics. Superimposition of sequential molar distalization of upper teeth, frames extracted by ClinCheck (Align Technology, Inc., San Jose, CA, USA); the sequence of movements was distal crown tipping followed by distal root uprighting

**Fig. 2:**
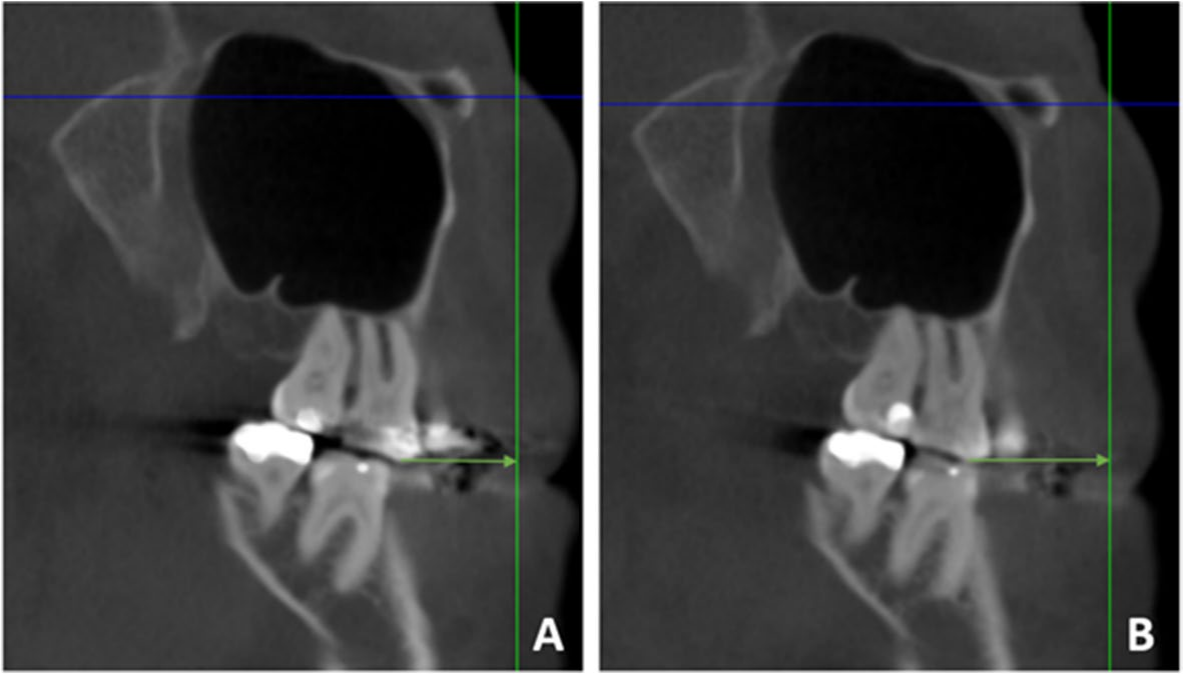
Distance between coronal plane and mesiobuccal cusp of maxillary first molar at (T0) before distalization (**A**) and at (T1) after distalization (**B**)

Inclusion criteria: (1) Adult patients having full permanent dentition except third molars, with half cusp to full cusp class II molars relationship with non-extraction treatment protocol. (2) Cases with standardized treatment protocol for pure maxillary sequential molar distalization (no planned rotation, tip nor torque) supported by conventional attachments for aligners’ support. Sequential molar distalization was achieved as follows: maxillary second molars were distalized first and once reached half the way, the first molars were moved back. When the first molars moved midway, the second molar arrived at the planned position, then the premolars move and so on [12]. This distal movement was supported by Class II elastics (3/16 − 3.5 ounces) from a cutouts on upper canines to lower first molars and the patients were instructed to wear them full time (Fig. 1). (3) Cases with a minimum of 2 mm actual maxillary first and/or second molar(s) distalization (as measured on CBCT) (Fig. 2). (4) The cases that were chosen for the trial met the compliance requirements (as documented in their files), which included wearing their aligners at least 22 h per day as advised by Align Technology, with regular 6-week monitoring for encouragement. (5) Absence of maxillary third molars on CBCT (6) Absence of maxillary deficiency. Exclusion criteria: Previous orthodontic treatment, cases with transverse deficiency and history of systemic disease, craniofacial syndromes, or presence of cleft lip or palate.

This study was approved by the Institutional Review Board at the Faculty of Dentistry, Alexandria University, Alexandria, Egypt (IRB:00010556–IORG:0008839). Manuscript Ethics Committee number (0423-04/2022). All records were of patients who consented for the use of records for research or educational purposes following the ethical approval at University of Alberta, Alberta, Canada protocol #(Pro00091339). All records were de-identified before being enrolled in the study. All methods were carried out in accordance with the Declaration of Helsinki. Informed consent was obtained from all subjects/or their legal guardian(s) for the use of their records. Neither minors nor illiterates were included in this study.

### CBCT image analysis

**Fig. 3:**
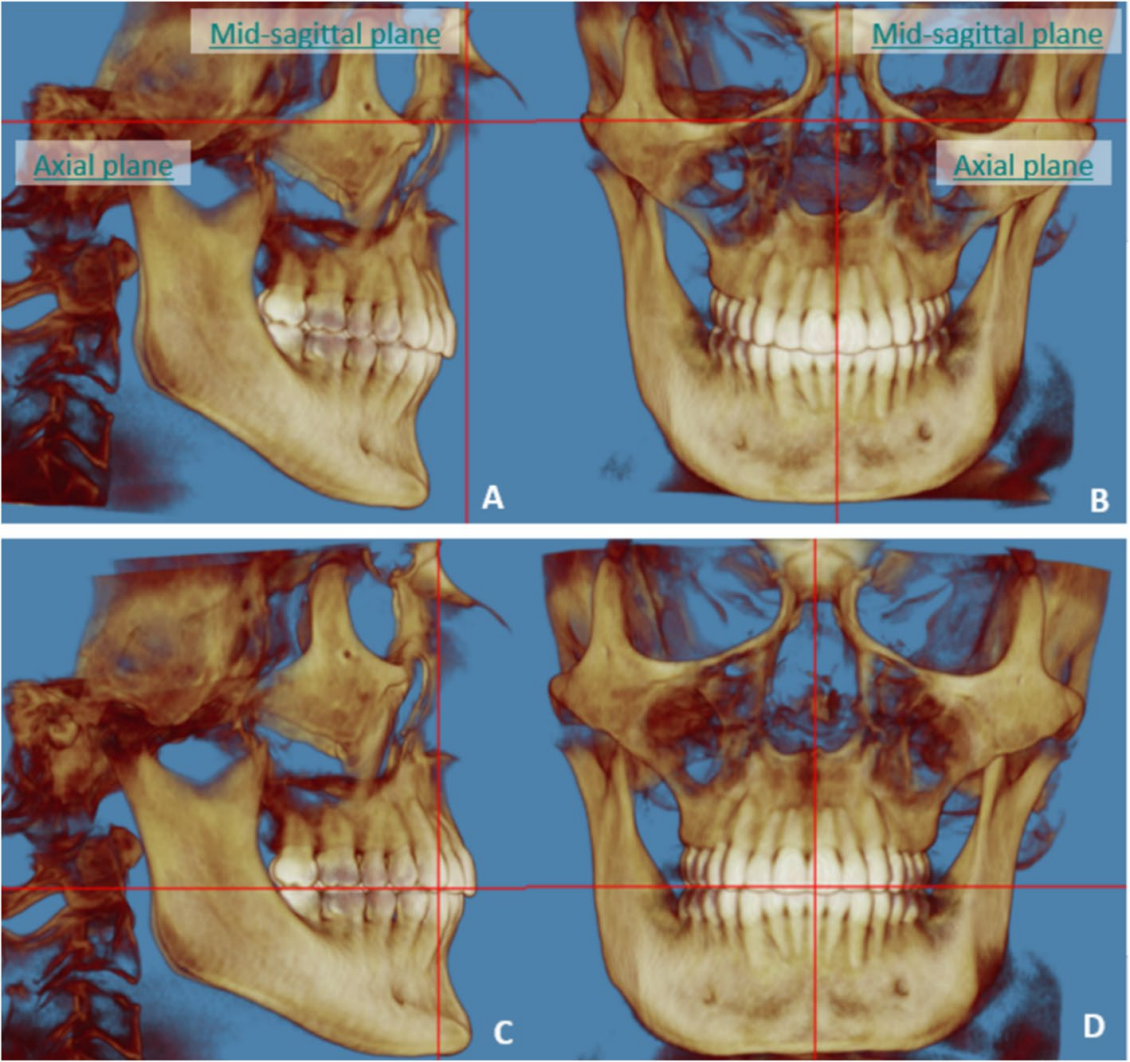
CBCT images were oriented with the midsagittal plane aligned to nasion perpendicular (**A**-**D**) and the axial plane aligned to either FHP (**A**-**B**) or occlusal plane (**C**-**D**) to consistently measure the selected parameters that are related to each orientation

All digital CBCT images acquired before and after orthodontic treatment using clear aligners were analyzed using Dolphin Imaging software version 11.95 Premium (Dolphin Imaging, Chatsworth, CA). Each CBCT image was set up with the mid-sagittal plane perpendicular to Nasion in the coronal and sagittal views. Each CBCT was oriented twice: First orientation, by setting the axial plane through the Frankfort horizontal plane on the sagittal view. This was used to measure the amount of molar distalization and molar angulation (mesiodistal tipping) (Fig. 3, A-B). Two sagittal sections were used, one showing the mesiobuccal (MB) and distobuccal (DB) root apices, and another one showing the palatal (Pa) root apex. Coronal slices were used to measure the molar inclination (Bucco-lingual torque). In the second orientation, the axial plane was reoriented passing through the occlusal plane to measure the amount of molar rotation (Fig. 3, C-D). Measurements were performed by the same investigator using measurement tool in Dolphin Imaging software (Dolphin Imaging, Chatsworth, CA).

### Maxillary molar distalization

CBCT-driven views in the sagittal plane were used to evaluate the amount of maxillary molar distalization. Linear measurements were made from the mesiobuccal cusps of the maxillary first molar and second molar to the coronal plane (respectively), at T0 and T1 (Fig. 2).

### Molar angulation and inclination

**Fig. 4:**
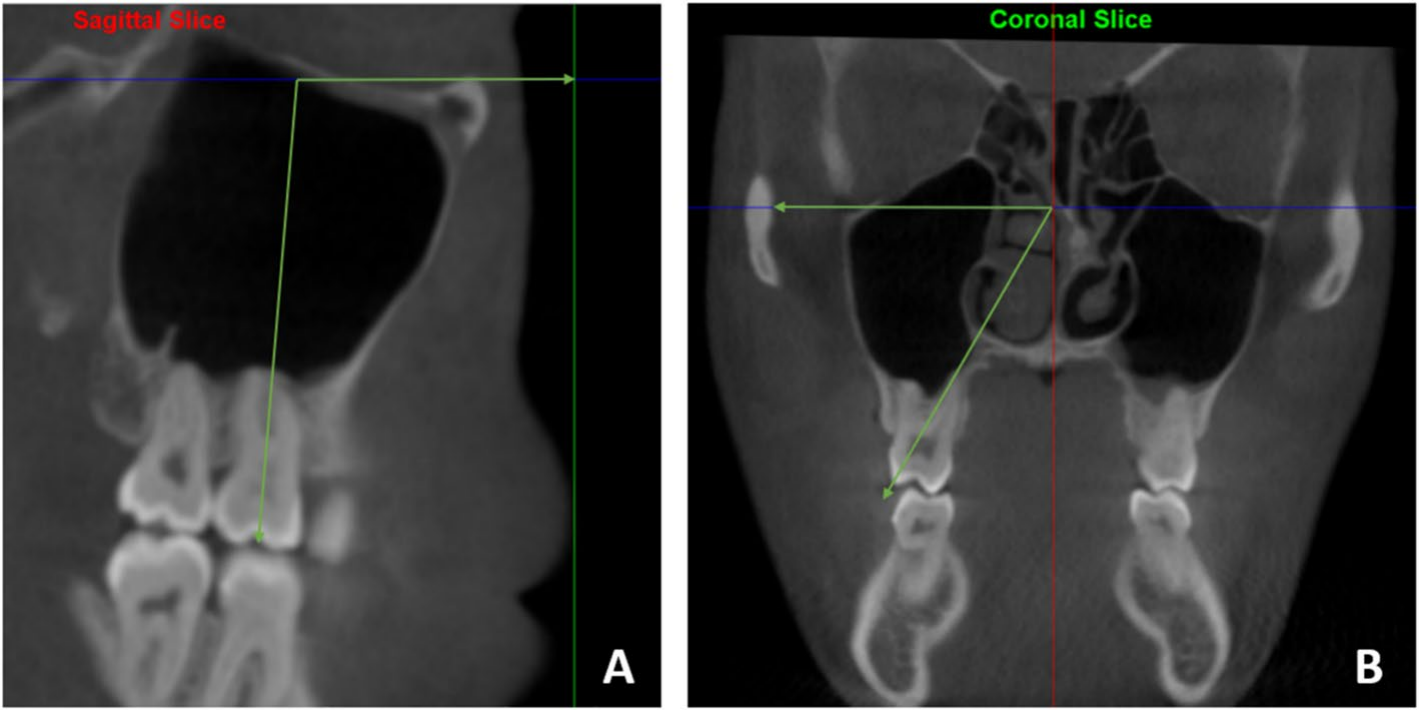
Measurement of maxillary first permanent molar angulation (**A**) and Inclination (**B**)

Molar angulation (mesiodistal tipping) was measured on the sagittal sections of the pre- and post-treatment CBCTs as the angle between the long axis of the maxillary molar (i.e., the line passing through molar bifurcation and Frankfurt horizontal plane (FHP). On the coronal sections, molar inclination (buccolingual inclination) was measured as the angle between a line connecting the buccal cusp tip and the palatal root apex and the FHP (Fig. 4).

### Molar rotation

**Fig. 5:**
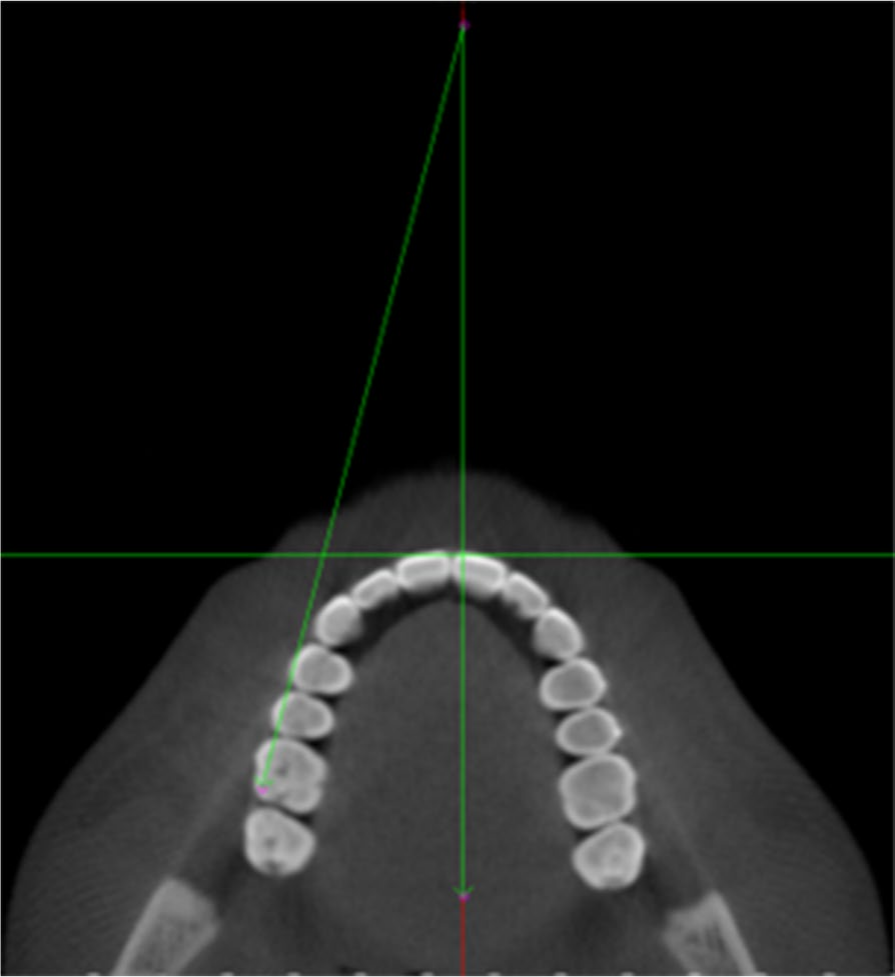
Molar rotation measurement on axial view

To measure molar rotation, the CBCT orientation was modified, with the axial plane passing through the occlusal plane (Fig. 3, C-D). On the axial images, molar rotation was measured as the angle between the line joining the MB and DB cusps and the mid-sagittal plane (Fig. 5).

### Statistical analysis

Statistical analysis was carried to study the effect of maxillary molar distalization using aligners on the molar angulation, inclination and rotation. Ten CBCTs were used to calibrate the investigator before conducting the study. The measurements were repeatedly conducted until an acceptable level of agreement is achieved. All parameters were measured twice by the same examiner one week apart, to assess intra-rater reliability. Also, a second examiner remeasured ten random cases for assessing inter-rater reliability. This was evaluated using summary statistics for the differences between the repeated measurements and intraclass correlation coefficients (ICCs). ICC values higher than 80% indicate excellent reproducibility [16]. Quantitative data were described using range (minimum and maximum), mean and standard deviation. The Kolmogorov-Smirnov and Shapiro-Wilk tests were used to verify the normality of distribution, Thus, differences between pre- and post-treatment readings were evaluated using paired t-test, whereby *p* ≤ 0.05 indicated statistical significance.

## Results

The ICC for intra- rater reliability showed excellent reproducibility for linear measurements (0.94–0.99) and angular measurements (0.86–0.99). Excellent inter-rater reliability (above 0.9) for all measurements was observed [16]. Molar angulation did not show significant change after distalization (from 95.77 ± 7.25**°** to 94.88 ± 6.71**°**) (*p* = 0.158), there was a significant increase in molar inclination (buccal crown torque) (from 59.0 ± 5.92**°** to 57.32 ± 6.22**°**) (*p* = 0.034). The molars elicited significant mesiobuccal rotation as evidenced by the angle between the line joining the MB and DB cusps and the mid-sagittal plane on the axial cut, decreasing from 15.12 ± 6.25**°** to12.89 ± 5.17**°** (*p* < 0.001) (Table 1).

**Table 1:** Comparison between mean initial (T0) and final (T1) angulation, inclination, and rotation of maxillary molars (*n* = 61)

	Initial (T0)	Final (T1)	Change (T1-T0)	t	*p*
**Angulation ° (Tipping)**					
Min. – Max.	79.70–110.60	75.10–109.60	-13.0–13.40 0.89 ± 4.85	1.430	0.158
Mean ± SD.	95.77 ± 7.25	94.88 ± 6.71			
**Inclination (Torque)**					
Min. – Max.	47.90–73.30	41.40–72.0	-20.30–19.40 1.67 ± 6.01	2.172^*^	0.034^*^
Mean ± SD.	59.0 ± 5.92	57.32 ± 6.22			
**Rotation**					
Min. – Max.	2.10–32.70	0.90–25.0	-4.90–15.20 2.23 ± 3.93	4.431^*^	< 0.001^*^
Mean ± SD.	15.12 ± 6.25	12.89 ± 5.17			

*SD* Standard deviation, *t* Paired t-test, *p* *p* value for comparing between Initial and Final

*Statistically significant at *p* ≤ 0.05

When the first and second molars movements were analyzed separately the same pattern of movement was elicited. Maxillary first molars did not show significant change in molar angulation with (*p* = 0.70), however showed a significant increase in molar inclination and mesiobuccal rotation with (*p* < 0.001 and *p* = 0.03) respectively (Table 2). Maxillary second molars did not show significant change in molar angulation with (*p* = 0.10), however showed a significant increase in molar inclination and mesiobuccal rotation with (*p* = 0.1 and *p* = 0.001) respectively (Table 3).

**Table 2:** Comparison between mean initial (T0) and final (T1) angulation, inclination, and rotation of maxillary first molars (*n* = 30)

	Initial (T0)	Final (T1)	Change (T1-T0)	t	*P* value
Angulation ° (Tipping)	92.80 ± 5.68	92.43 ± 5.55	-0.37 ± 5.22	0.39	0.70
Inclination (Torque)	60.80 ± 5.65	57.31 ± 4.70	-3.49 ± 4.81	4.04	< 0.001*
Rotation	16.64 ± 5.59	15.24 ± 3.75	-1.40 ± 3.32	2.36	0.03*

*SD* Standard deviation, *t* Paired t-test, *p* *p* value for comparing between Initial and Final

*Statistically significant at *p* ≤ 0.05

**Table 3:** Comparison between mean initial (T0) and final (T1) angulation, inclination, and rotation of maxillary second molars (*n* = 31)

	Initial (T0)	Final (T1)	Change (T1-T0)	t	*P* value
Angulation ° (Tipping)	98.84 ± 7.50	97.42 ± 6.94	-1.42 ± 4.45	1.75	0.10
Inclination (Torque)	59.14 ± 6.91	56.65 ± 7.10	-2.48 ± 5.15	2.64	0.01*
Rotation	13.54 ± 6.59	10.46 ± 5.36	-3.08 ± 4.37	3.86	0.001*

*SD* Standard deviation, *t* Paired t-test, *p* *p* value for comparing between Initial and Final

*Statistically significant at *p* ≤ 0.05

## Discussion

Maxillary molars distalization provides a valid treatment modality for several malocclusions, including Class II cases and crowding [7, 12, 13]. Today, molar distalization can be carried out using aligners, which have been demonstrated to be efficient when a mean of 2.25–2.6 mm molar distalization movement is required [11, 17]. The actual 3D maxillary molar positional changes during distalization using Invisalign i.e. molar angulation (mesiodistal tipping), inclination (buccolingual torque) and rotation is not yet fully documented, as many studies agree such testing requires comparing CBCT before and after treatment [18, 19]. Only a single recently published study addressed this question, but only using digital models and clincheck [20].

Two-dimensional (2D) linear measurements derived from 3D images were used in this work based on several recent studies [21–23]. Recent publications reported that both 3D and 2D superimpositions are considered highly reliable [24, 25], as they did not elicit significant difference in measurements.

Cases included in the study had an actual 2 mm of pure molar distalization as assessed from CBCT not from digital models nor clincheck prediction. Since it was proven that predicted molar distalization is overestimated than the actual amount. Liu et al. [26] found that the efficacy of posterior teeth distalization was only 36.2–43.9% after the entire arch was distalized using clear aligners, regardless of whether class II elastics or intramaxillary miniscrews were used for anchorage control. Furthermore, Li et al. [27] reported 0.78 mm and 0.99 mm of distalization for maxillary first and second molars respectively with anterior retraction; this indicates an efficacy of 31.50% for the first molar and 35.63% for the second molars distaization by using clear aligners.

The current study showed that molars can be distalized by 2 mm using Invisalign without eliciting significant tipping. This is in agreement with the findings of Ravera et al. [11] who concluded that distal displacement of 2.25 mm and 2.52 mm for maxillary first and second molars (respectively) can be achieved without significant molar tipping using clear aligners. Furthermore, Simon et al. [28] illustrated from scanned casts that distal molar displacement of 2–3 mm can be achieved without significant molar tipping using CAT. A recent study by Cui et al. [29] studied the morphology changes of maxillary molar during CAT distalization and concluded that the first and second molar showed a translation movement without significant tipping. Clear aligners seem to control molar tipping during distalization more than other conventional orthodontic appliances as Jones jig, Pendulum, and distal jet [7]. This could be related to the difference in aligners design allowing the control of 3D movements by holding teeth on all the surfaces (vestibular, palatal-lingual, and occlusal), and applying appropriate levels of force, thanks to the digitally planned attachments. Conventional rectangular and optimized vertical attachments located on the buccal aspect of the distalizing molars and premolars respectively, did create a sufficient moment to oppose the undesirable tipping movement [30–32].

The ability to evaluate molar inclination (torque), which could only be performed on the coronal section of 3D-CBCT, was one of the strengths of the present study. There was significant buccal crown inclination during maxillary molar distalization using Invisalign with a mean of 1.67 degrees. Buccal crown torque increase was an expected finding during molar distalization, as it occurs even with fixed appliance therapy. Kinzinger et al. [12] reported buccal crown inclination during bilateral maxillary molar distalization using a modified Pendulum appliance in adolescent patients with an increase in intermolar distance of 2.11 ± 1.67 mm between mesiobuccal cusp tips. Moreover, Bolla et al. [7] also showed 2.9 mm of intermolar width increase with distal jet distalization. This is explained by the finite element analysis for upper molar distalization, which proved that bodily distal upper molar movement could be obtained only when rotational axis is at infinite, and the compressive stress is homogeneously distributed in the periodontal ligament [33].

There was significant mesiobuccal crown rotation during maxillary molar distalization using Invisalign, with a mean of 2.23° Mesiobuccal rotation similarly occurs during molar distalization with fixed appliances combined with Distal jet, Pendulum appliance, modified Pendulum and Jones jig [12, 14]. Thus, in cases requiring maxillary molar distalization using clear aligners, we suggest adding buccal root torque and rotational control attachments to control molars buccal flaring and rotation.

## Conclusions

1. Maxillary molar distalization of 2 mm did not cause significant molar tipping.
2. Maxillary molars showed significant buccal inclination (increased torque) and mesiobuccal rotation during distalization.

## Data Availability

Data sets used and/or analyzed during the current study are available from the corresponding author on reasonable request.

## Abbreviations

CAT: Clear aligner therapy
2D: Two-dimensional
3D: Three-dimensional
CBCT: Cone beam computed tomography
MB: Mesiobuccal
DB: Distobuccal
Pa: Palatal
FHP: Frankfurt horizontal plane
ICC: Intraclass correlation coefficient
